# *Clostridioides difficile*: innovations in target discovery and potential for therapeutic success

**DOI:** 10.1080/14728222.2021.2008907

**Published:** 2021-12-02

**Authors:** Tanya M Monaghan, Anna M Seekatz, Benjamin H Mullish, Claudia C. E. R Moore-Gillon, Lisa F. Dawson, Ammar Ahmed, Dina Kao, Weng C Chan

**Affiliations:** a NIHR Nottingham Biomedical Research Centre, University of Nottingham, Nottingham, UK;; b Nottingham Digestive Diseases Centre, School of Medicine, University of Nottingham, Nottingham, UK;; c Biological Sciences, Clemson University, Clemson, SC, USA;; d Division of Digestive Diseases, Department of Metabolism, Digestion and Reproduction, Faculty of Medicine, Imperial College London, London, UK;; e Departments of Gastroenterology and Hepatology, St Mary’s Hospital, Imperial College Healthcare NHS Trust, London, UK;; f Department of Infection Biology, London School of Hygiene and Tropical Medicine, London, UK;; g Department of Gastroenterology, Zeidler Ledcor Centre, University of Alberta, Edmonton, Alberta, Canada;; h School of Pharmacy, Biodiscovery Institute, University of Nottingham, University Park, Nottingham, UK

**Keywords:** *Clostridioides difficile* infection, new drug targets, small inhibitors, therapeutics, toxins, virulence factors

## Abstract

**Introduction:**

*Clostridioides difficile* infection (CDI) remains a worldwide clinical problem. Increased incidence of primary infection, occurrence of hypertoxigenic ribotypes, and more frequent occurrence of drug resistant, recurrent, and non-hospital CDI, emphasizes the urgent unmet need of discovering new therapeutic targets.

**Areas covered:**

We searched PubMed and Web of Science databases for articles identifying novel therapeutic targets or treatments for *C. difficile* from 2001 to 2021. We present an updated review on current preclinical efforts on designing inhibitory compounds against these drug targets and indicate how these could become the focus of future therapeutic approaches. We also evaluate the increasing exploitability of gut microbial-derived metabolites and host-derived therapeutics targeting VEGF-A, immune targets and pathways, ion transporters, and microRNAs as anti-*C. difficile* therapeutics, which have yet to reach clinical trials. Our review also highlights the therapeutic potential of re-purposing currently available agents . We conclude by considering translational hurdles and possible strategies to mitigate these problems.

**Expert opinion:**

Considerable progress has been made in the development of new anti-CDI drug candidates. Nevertheless, a greater comprehension of CDI pathogenesis and host-microbe interactions is beginning to uncover potential novel therapeutic targets, which can be exploited to plug gaps in the CDI drug discovery pipeline.

## 1. Introduction

*Clostridioides difficile* is a gram positive, strictly anaerobic, spore-forming bacterium capable of colonizing the gastrointestinal tract, and a leading worldwide cause of antibiotic-associated and healthcare-acquired infectious diarrhea [1]. Although the majority of *Clostridioides difficile* infection (CDI) cases are triggered by antibiotic exposure, other risk factors include older age, immunosuppression, inflammatory bowel disease, and chronic kidney disease [2–4]. The epidemiology of *C. difficile* is evolving, with increasing reports of community-acquired *C. difficile* infection (CDI) in persons who are young and otherwise healthy [5]. The clinical spectrum ranges from asymptomatic carriage, mild infection with diarrhea to toxic megacolon and multiorgan failure in fulminant cases [2], where attributable mortality rates range between 30% and 60% [6].

Interruption of the gut the intestinal microbial composition, commonly referred to as microbial dysbiosis, to predominance by Proteobacteria rather than Firmicutes and Bacteroidetes is integral to CDI pathogenesis [7]. Once established in the gut, *C. difficile* produces the virulence factors TcdA and TcdB, toxins belonging to the larger clostridial glycosylating toxin family, with TcdB being more virulent [8]. Furthermore, adhesion and motility-associated factors, such as surface layer protein (SlpA), cell wall protein 84 (Cwp84), flagellar components, and the presence of *C. difficile* binary toxin CDT, all play roles in CDI pathogenesis [9,10]. Although certain ribotypes, such as 027 and 078, are associated with severe infection [11–13], determinants of disease severity and recurrence risk are largely shaped by the complex host immune response to CDI, much of which remains poorly understood. For example, anti-toxin antibodies play a protective role and prior studies have shown that low serum anti-toxin A and B antibody levels are associated with an increased risk of recurrent CDI [14,15]. However, when used in combination with standard-of-care antibiotics, only Bezlotoxumab, a fully human anti-toxin B monoclonal antibody, not Actoxumab, a monoclonal anti-toxin A antibody, is effective in preventing CDI recurrence in clinical trials [16].

The mainstay of treatment for CDI is antibiotics, which may worsen dysbiosis, contributing to CDI recurrence. The conventional first-line therapy for CDI is vancomycin, a glycopeptide antibiotic targeting mainly Gram-positive bacteria. An alternative is fidaxomicin, a narrow spectrum macrolide antibiotic, which is superior to vancomycin in preventing CDI recurrence, although it is not currently commonly used as first-line therapy due to its high cost. Although the Infectious Diseases of America (IDSA) and Society for Healthcare Epidemiology of America (SHEA) recently recommended the use of fidaxomicin rather than standard course of vancomycin as the preferred therapy for an initial episode of CDI, its implementation will clearly depend upon available resources [17]. Metronidazole may still be used in a primary episode in younger patients with mild to moderate infection, although resistance is increasing [18].

Following treatment for the initial episode of CDI, approximately 15% to 30% of patients may experience CDI recurrence, and the risk continues to rise with each subsequent episode, reaching approximately 45% to 65% after the third episode [19]. Since conventional therapeutic options are limited, managing patients with recurrent CDI (rCDI) is a major clinical challenge. Thus, better and more targeted treatments for the initial episode of CDI is key to preventing rCDI. Pulsed tapered vancomycin is the usual treatment for rCDI. Bezlotoxumab has demonstrated modest efficacy in preventing CDI recurrence when used as an adjunct to CDI directed antibiotics, but due to high cost, its role in the rCDI treatment algorithm remains unclear. On the other hand, fecal microbiota transplantation (FMT) has shown very high efficacy in this patient population [20]. However, FMT is an untargeted therapy, and the risks of potential infectious transmission cannot be overlooked [21,22]. Defined microbial consortium would negate such risks. Recently, Microbial Ecosystem Therapeutics (MET-2) was shown in a small open-label trial of 19 patients to have similar efficacy to FMT [23]. Although the precise mechanisms of FMT or live microbial therapeutics are not well understood, several mechanisms have been shown to mediate the resolution of recurrent CDI [24,25].

**Figure 1.**
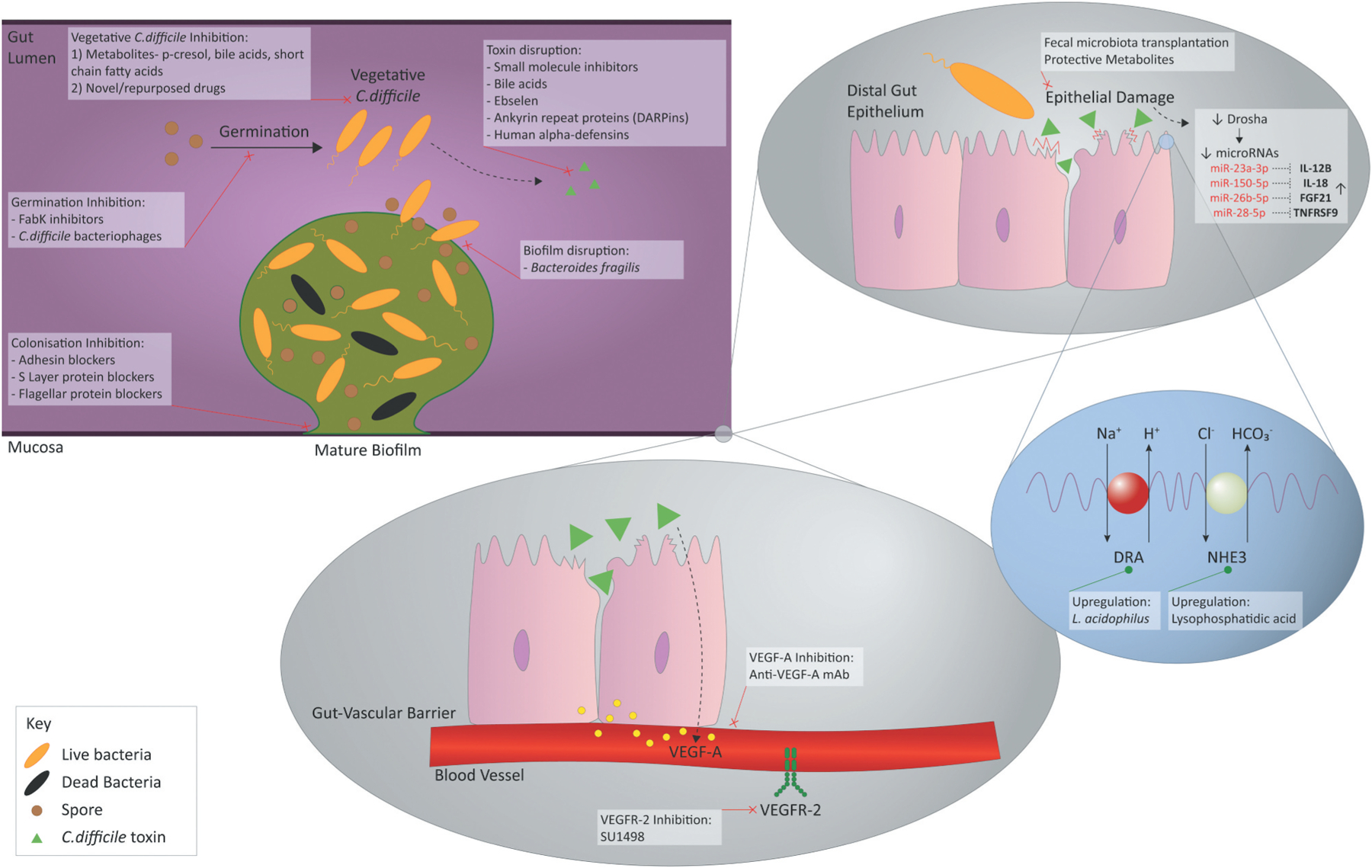
Emerging therapeutic targets and treatment strategies against *Clostridioides difficile*.

In this review, we highlight new potential therapeutic targets for CDI treatment, examining different strategies. These range from repurposed drugs aimed at *C. difficile* to new precision therapeutics that target *C. difficile* virulence factors, as well as and precision immune- and epigenetic therapeutic approaches that modulate the host immune and inflammatory response (Figure 1). We also describe the perceived challenges in bringing these new approaches to the clinic.

## 2. Novel therapeutic strategies

### 2.1. *C. difficile* adhesins

Several adhesins on both the spore and vegetative cell types of *C. difficile* represent potential pathogenic targets to attenuate colonization, virulence, and recurrence. This section will review some of the recent advances made in targeting inhibition of *C. difficile* adhesion to host cells.

Recent evidence suggests that binding by exosporium proteins to mucin glycoproteins is key to initial colonization, involving both binding and internalization of the spore to the intestinal epithelium. *In vitro* binding studies demonstrated that CotE, a peroxiredoxin-chitinase protein, mediated binding to the mucin glycoproteins, as well as the monomers GlcNAc and GalNAc [26]. Infection with strains lacking CotE also resulted in increased survival in a hamster model of disease. The collagen-like protein BclA1 have previously been demonstrated to aid binding to the epithelial layer [27]. A recent study extended this observation, demonstrating *in vitro* that BclA3 mediates binding and internalization of the spore via the host proteins fibronectin and vitronectin [28]. Mice infected with a BclA3 mutant strain demonstrated reduced recurrence rates, suggesting that spore adhesion could contribute to the persistence of spores in the gut during antibiotic treatment. Blocking exosporium protein binding may thus represent a therapeutic target to not only decrease initial colonization of *C. difficile* but also prevent recurrence. Indeed, preliminary studies suggest that BclA3 epitopes can induce anti-spore IgG antibodies in a mouse model of infection, highlighting new vaccine targets to inhibit *C. difficile* entry and persistence [29].

The cell wall of *C. difficile* incorporates several surface layer (S-layer) proteins (SLPs) that promote adhesion and growth of vegetative cells. The *C. difficile* conserved gene *slpA* encodes for two SLPs, a high molecular weight (HMW-SLP) and low-molecular weight (LMW-SLP), that are major mediators of binding to the epithelium [30]. Pre-treatment of cells with purified SlpA or anti-slpA sera has been demonstrated to reduce *C. difficile* attachment to host cells *in vitro* [31]. Partial protection has been observed previously in mice vaccinated with SlpA [32]. More recently, administration of lactic acid bacteria that also express SLP orthologs engineered to express *C. difficile* SLPs could prevent CDI-induced death in a hamster model of infection [33].

The flagellar proteins fliC and fliD are involved in attachment to the epithelial layer, thus representing another target to block colonization [34]. Hamsters challenged with FliC protein-encapsulated pectin beads were partially protected from *C. difficile* challenge and exhibited anti-FliC IgG antibodies following intra-peritoneal immunization, supporting the addition of flagellar epitopes to vaccine candidates that target other virulence aspects [35]. Recent findings demonstrate that flagellar expression is also phase variable, inducing swimming motility and higher toxin production [36]; however, considerable variation is likely across different *C. difficile* ribotypes, complicating the clinical implications of targeting flagella for immunization [37].

### 2.2. Biofilms

Like many pathogens, *C. difficile* has been shown to form biofilms in the gastrointestinal tract of mice [38] and hamsters [39], as well as in multispecies biofilms with representatives of the human gut microbiome [40]. The formation of biofilms has the potential to promote persistence and resistance to antibiotics [41,42]. *C. difficile* genomes contain a *luxS* gene homolog, which encodes for a common signaling molecule, autoinducer-2, involved in quorum sensing to initiate biofilm formation and transcription of virulence factors [43]. In addition to LuxS, flagella, the cell wall protein Cwp84, and the master regulator of sporulation SpoA have been involved in biofilm formation in vitro [41,44,45]. Biofilms form a protective shield around vegetative cells and spores which confers resistance to vancomycin [41,42] and oxygen [46]. Extracellular DNA and spore frequency has also been associated with biofilm formation, further demonstrating the importance of adhesion and sporulation proteins in biofilms [42]. Although the role of biofilm formation remains unclear during *in vivo* infection, a recent study demonstrated increased growth and germination when *C. difficile* was encased in a biofilm compared to *C. difficile* spores in a chemostat model consisting of pooled human feces [40]. Interference with biofilm formation, or disassembling established biofilms with DNase treatment [42], as well as related mechanisms like sporulation, could thus render *C. difficile* more susceptible to antibiotic therapies and/or prevent persistence of spores to reduce recurrence.

More recently, there is interest in targeting mixed bacterial populations to prevent *C. difficile* biofilm formation and thus persistence in the gut. *Bacteroides fragilis*, a gut commensal microbe that has been negatively associated with *C. difficile* during infection, was demonstrated to inhibit *C. difficile* biofilm formation *in vitro* in co-culture [44]. The presence of LuxS was observed to contribute to *C. difficile* growth inhibition in this co-culture [44], suggesting different metabolic and signaling pathways for mono- vs mixed-populations. In a bioreactor model consisting of human feces, *C. difficile* has been observed to co-colonize with *Fusobacterium nucleatum*, another prevalent human gut commensal bacterium [47]. Aggregation between the two was diminished when *C. difficile* flagella or the RadD protein of *F. nucleatum* was removed. Targeting of non-*C. difficile* factors, such as proteins of other bacteria that interact with *C. difficile* directly, could be a unique therapeutic landscape.

### 2.3. *P*-Cresol production

An unusual feature of *C. difficile* among the gut microbiome is its ability to produce high concentrations of the antibacterial compound *para*-cresol. In *C. difficile, p*-cresol production occurs either through the fermentation of tyrosine via the intermediate *para*-hydroxyphenylacetate (*p*-HPA) [48], or via turnover of exogenous *p*-HPA [49] present in the human gut. This decarboxylation of *p*-HPA to *p*-cresol is performed by the *HpdBCA* decarboxylase encoded by the *hpdBCA* operon [48]. Dawson et al have shown that each subunit (*hpdB, hpdC* and *hpdA)* is essential for *p*-cresol production [50]. The production of *p*-cresol by *C. difficile* has a deleterious effect on the gut microbiome, promoting dysbiosis, by targeting the Gram-negative cell envelope, which confers a fitness advantage over other intestinal bacteria and enhances relapse of CDI [51]. *C. difficile* is only one of four gut bacteria (*Blautia hydrogenotrophica, Romboutsia lituseburensis* and *Olenella uli*) capable of high-level *p*-cresol production (>100 μM), with an additional 55 gut bacteria capable of producing <10 μM [52]. However, the *hpdBCA* operon is highly conserved in all the sequenced *C. difficile* isolates [51], and interestingly only the three other high *p*-cresol producing gut bacteria had orthologues to the *hpdBCA* operon (>30% amino acid sequence identity). Interestingly, the hypervirulent strain R20291 produced significantly more *p*-cresol than the 630 strain and is more tolerant to *p*-cresol [53]. Regulation of *p*-cresol production involves induction of the *HpdBCA* decarboxylase via the presence of *p*-HPA, in a concentration dependant manner [49], indicating that sensing and responding to *p*-HPA is key to *p*-cresol production. The *p*-cresol biosynthesis pathway, and more specifically the *HpdBCA* decarboxylase, is an alternative druggable target, which would have minimal perturbations on the healthy gut microbiome and thereby reduce the potential off target effects of broad-spectrum antibiotics that promote dysbiosis.

Detection of *p*-cresol could also enhance diagnosis of CDI. The presence of *p*-cresol can be detected by gas liquid chromatography using a portable Z-Nose [53] and has been shown to be present in stool samples of mice infected with *C. difficile* [51]. The Z-Nose is an ultra-rapid analytical device which allows the real-time monitoring of volatile compounds [54], such as *p*-cresol [53]. The detection of *p*-cresol in stool samples at point-of-care would potentially speed up diagnosis and treatment of CDI.

### 2.4. Phage-based therapeutics

Bacteriophage are bacterial viruses, which can infect and kill certain bacteria. Phage have a narrow host range, which makes them a potentially useful therapeutic to target specific pathogenic bacteria [55]. There are main two types of phage, lytic and temperate. Temperate phage have the capacity to be either lytic or lysogenic depending on the host and environmental conditions, however only lytic phage show therapeutic potential. To date, a limited number of *C. difficile* specific lytic phage have been identified. Phage produced endolysins/lysins rapidly degrade cell wall peptidoglycan to facilitate release of bacteriophage progeny following replication [56]. Bacteriophage-derived endolysins and their derivatives have shown efficacy as a novel class of antibacterial agents. *C. difficile* bacteriophages produce amidase and endolysin. Mondal et al. [57] identified that recombinantly expressed cell wall hydrolase (CWH) lysin from *C. difficile* phage, phiMMP01, was active against *C. difficile* and this activity could be enhanced by removing the N-terminal cell wall binding domain, creating CWH351 – 656 [57]. Interestingly, Mayer et al. [58], identified that truncation of the endolysin CD27L from phage ΦCD27, retaining just the N-terminal domain, CD27L1–179, increased lytic activity against *C. difficile* [58]. This recombinant CWH (CWH351 – 656) inhibits spore outgrowth *in vitro*, which the authors suggest is critical in preventing the spread and recurrence of *C. difficile* infection [57]. Phage lytic enzymes have been shown to achieve a 4-log reduction in *C. difficile* vegetative cell viability in 5 hours after external application [59], potentially making them a narrow spectrum target for CDI. However, the lack of naturally occurring strictly lytic phages in *C. difficile* has significantly impacted phage therapy [60], but the use of recombinant lytic phage protein has opened a door for targeted phage therapy.

### 2.5. FabK inhibitors

Fatty acids play a crucial role in the maintenance of the integrity of bacterial cell membranes, and their biosynthesis, as part of the non-mammalian type II fatty acid synthase (FASII) pathway, is mediated by a multitude of interlinked acyl carrier proteins (ACPs) in the cytoplasm [61,62]. Substrates of this pathway are bound to ACPs such as FabD, FabF and FabG, and undergo a series of reactions to extend their acyl chains; each elongation cycle is culminated by reduction, which is catalyzed by an enoyl-acyl carrier protein reductase. These enzymes thus serve as suitable antimicrobial targets, an example of which is isoniazid, an antibiotic used in treating *Mycobacterium tuberculosis*, and one which functions by inhibiting the reductase FabI [63]. Since bacterial species utilize specifically distinct enoyl-ACP reductases, its inhibition additionally provides a selective antimicrobial target. For instance, triclosan, another known FabI inhibitor, does not inhibit reductases such as FabK and FabV [64,65]. Although not as extensively studied as FabI, FabK has previously been reported as the sole reductase present in *Streptococcus pneumoniae* [66], and this has led to the identification of FabK inhibitors [67], including compounds derived from phenylimidazole [68].

Over the last few years, FabK has been found to be particularly noteworthy in the context of CDI. Unlike almost all other gut bacteria, which utilize only FabI or both FabI and FabK, *C. difficile* has been shown to exclusively make use of FabK [69], thereby making it an ideal candidate for selective inhibition by FabK inhibitors, and concurrently limiting the wider damage to the gut. Indeed, a follow-up study has demonstrated considerable efficacy of a range of phenylimidazole-derived compounds in inhibiting proliferation of *C. difficile*, whilst having a minimal impact on other gut bacterial species [70]. The significance of targeting FabK in *C. difficile* is further highlighted by the fact that unlike current anti-CDI therapies, it not only limits sporulation (a key factor in disease recurrence), but given that the expression of FabK is itself controlled by the transcription factor FapR, its FASII system has been shown to resist being bypassed in the presence of exogenous lipids; the latter is in contrast to the interaction seen with most other gut bacterial species, where extracellular lipids in the gut are known to circumvent FASII, thus reducing the efficacy of any therapies that target this system [69]. Overall, FabK has demonstrated significant *in vitro* potential as a selective *C. difficile* target, and further scientific enquiry may allow FabK inhibitors to become a targeted treatment of CDI.

### 2.6. Toxin-targeted therapies

Targeting the highly pathogenic toxins of *C. difficile* represents a promising therapeutic approach to treat the symptoms associated with infection. Tam et al. [71] used chemical libraries composed of diverse small molecules consisting of natural products, drugs approved for human use, and molecules with known biological activity to identify 66 compounds capable of protecting cells from TcdB-induced rounding. These include toxin-modifying compounds, a toxin-stabilizing molecule (methyl cholate), inhibitors of endosomal acidification (bafilomycin A1, ammonium chloride, carboxylic ionophore monensin, aminacrine, amodiaquine, quinacrine, prazosin), and noncompetitive inhibitors of glucosyltransferase (GTD) activity (phloretin and its derivatives). In more recent work by the same authors [72], primary and secondary bile acids, which are known to play a role in *C. difficile* germination and outgrowth, were shown to directly modulate the structure and function of TcdB. Bile acid binding through the C-terminal combined repetitive oligopeptide repeats (CROP) domains, induces a major conformational change in TcdB structure that prevents receptor binding and uptake into cells. In follow on work, the team screened 2401 small molecules and identified 15 with a bile-acid-like mechanism of action. Alongside methyl cholate, they identified other hits including chenodeoxycholic acid, cholic acid, and lithocholic acid, betulinic acid, oleanoic acid, pregnanolone, asiatic acid as well as ethaverine [72]. Nonetheless, the therapeutic use of bile acids may be problematic due to the potential for overloading physiological functions of bile acids, particularly, signal transduction pathways and bile acid re-cycling processes. Of note, the microbiota is also altered after bile acid treatment [73].

**Figure 2.**
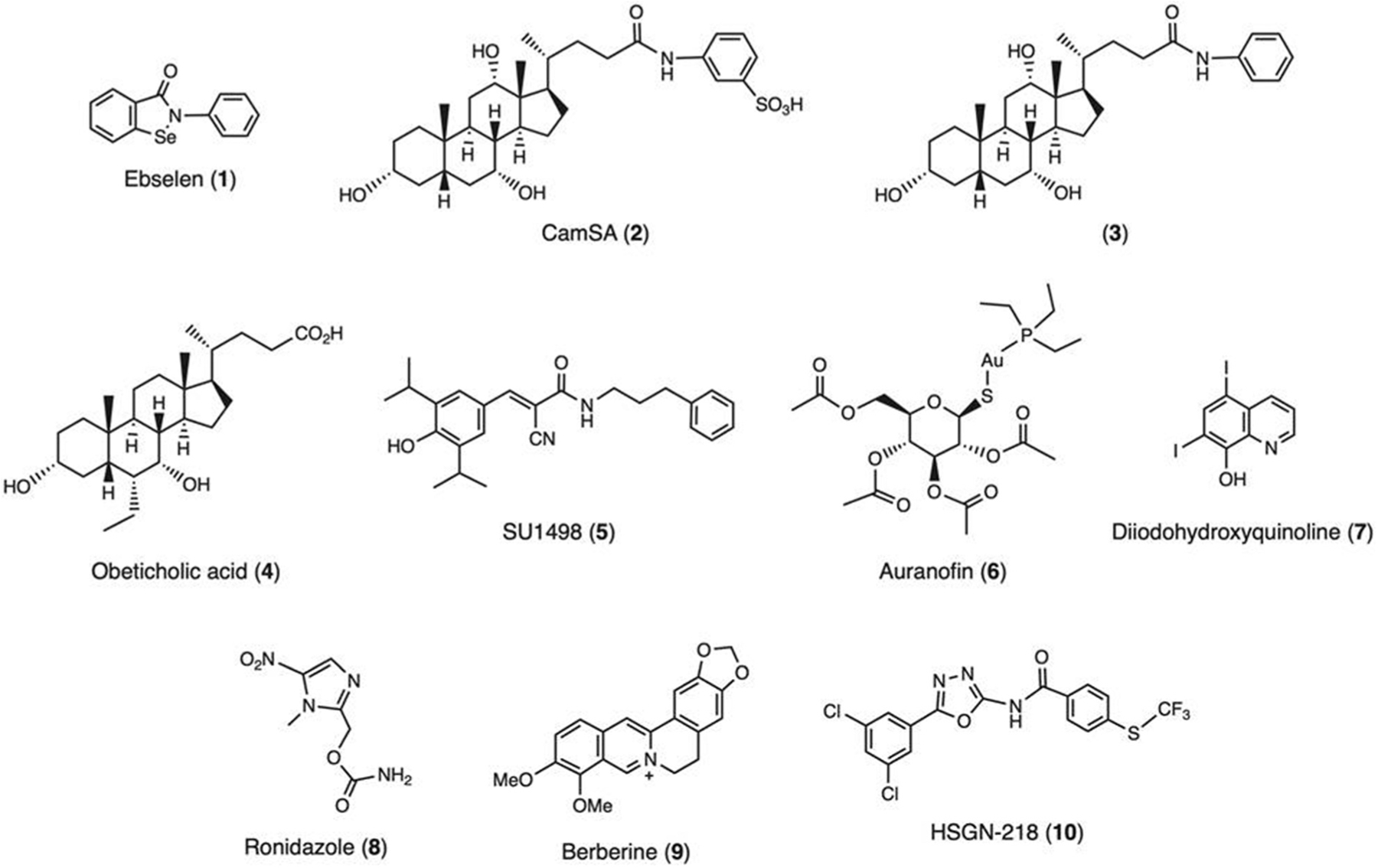
Examples of reported anti-*C. difficile* compounds.

Other small-molecule inhibitors of *C. difficile* toxins include ebselen (1; Figure 2), a synthetic organoselenium compound, work mechanistically through inactivation of TcdA [74] and TcdB via inhibiting the function of the auto-processing domain or the glucosyltransferase domain (GTD) [75].

Simeon et al. [76] report the TcdB-neutralizing capabilities of a series of monomeric and dimeric designed ankyrin repeat proteins (DARPins). Their findings show that DLD-4 displayed an approximately 330-fold higher potency than the commercially available anti-TcdB monoclonal antibody Bezlotoxumab in the same assay, and also protected mice from a toxin challenge in vivo. Competitive ELISA studies showed that the DARPins of DLD-4–1.4E and U3 interfere with the interaction between TcdB and its receptors chondroitin sulfate 4 (CSPG4) and frizzled class receptor 2 (FZD2), respectively. These highly potent DARPin-based antitoxins possess the potential to be developed into therapeutics to treat CDI and/or prevent its recurrence.

In addition to the large clostridial toxins TcdA and TcdB, a third *C. difficile* binary toxin (CDT) is associated with the most serious outbreaks of drug-resistance CDI. However, no therapeutic targeting strategies for CDT have been FDA-approved. New research has begun to elucidate the structure of the cell-binding component of CDT (CDTb), revealing a di-heptamer macromolecular assembly, and represents a starting point for developing structure-based drug-design strategies to target *C. difficile* [77].

Furthermore, new research has shown that defensins represent interesting therapeutic candidates to treat CDI. Defensins, as a prominent family of antimicrobial peptides (AMP), are major effectors of the innate immunity with a broad range of immune modulatory and antimicrobial activities. Typically, defensins promote local unfolding of the affected toxins upon binding, destabilize their secondary and tertiary structures, increase susceptibility to proteolysis, and leads to their precipitation [78]. Giesemann et al. [79] were the first to reveal that human alpha defensins human neutrophil protein (HNP)-1, HNP-3, and enteric HD-5 inhibited TcdB-induced cytotoxicity in multiple cell lines. These α-defensins inhibited the glucosylation of Rho proteins by toxin B. More recently, Korbmacher et al. [80] have shown that human antimicrobial peptide α-defensin-5 can efficiently neutralize CDT as well as TcdA and TcdB in cultured human intestinal epithelial cells. The same group of researchers has also reported that human α-defensin-1 protects cells and human intestinal organoids from the cytotoxic effects of TcdA, TcdB, CDT and their combination, and in mice, was also able to reduce TcdA-induced damage of intestinal loops in vivo [81].

### 2.7. Gut microbial metabolites: inhibitory role in sporulation and germination

Gut microbiota-derived metabolites impact upon numerous different aspects of metabolic and immune function within mammalian hosts [82]. Recognition that gut microbiota perturbation is a key contributor to CDI pathogenesis and that its restoration (i.e. via fecal microbiota transplant or FMT) is an efficacious treatment strategy, has fueled interest in the exploitability of gut microbial-derived metabolites as anti-*C. difficile* therapeutic agents [83].

Bile acids have been one particular focus, with interest deriving from the observation that different bile acids variably affect the ability of *C. difficile* to undergo vegetative growth or germination. Specifically, the primary conjugated bile acid, taurocholic acid (TCA), facilitates *C. difficile* spore germination [84], while secondary bile acids (including deoxycholic acid (DCA) limit the growth and toxin activity of *C. difficile* vegetative cells [85]. The biotransformation of primary to secondary bile acids *in vivo* is undertaken by enzyme systems produced by the gut microbiota but not mammals, and principally by two enzymes called bile salt hydrolase (BSH; which hydrolyses a glycine or taurine group from conjugated primary bile acids) and 7α-dehydroxylase (which converts unconjugated primary bile acids into secondary bile acids) [86]. Recurrent CDI patients develop gut enrichment of TCA but deficiency of secondary bile acids, which appears principally attributable to gut microbial BSH or 7α-dehydroxylase loss following prior antibiotic exposure [87–91]. Restoration of gut BSH functionality occurs after FMT for rCDI in humans [90], and delivery of 7α-dehydroxylase into rodent models is at least partially protective against CDI [92,93]. As such, the targeted administration of gut microbiota members with high bile-metabolizing functionality, or the administration of purified BSH and/or 7α-dehydroxylase, merits further consideration as an anti-CDI treatment strategy. There are several intuitive reasons that favor restoration of BSH-producing organisms as such a treatment approach compared to 7α-dehydroxylase-producers; for instance, BSH has relative insensitivity to oxygen and robust activity over a wide pH range, while 7α-dehydroxylase is a highly oxygen-sensitive enzyme [94]. An alternative possible strategy suggested by these data may be administration of exogenous secondary bile acids. However, such an approach would not remove pro-germinant bile acids (such as TCA) as an enzyme-based strategy would. Furthermore, excess intestinal secondary bile acids (including DCA) are recognized as a direct cause of colorectal tumorigenesis [95], and considerable care in dose titration would be required. An alternative proposed strategy may involve disruption of the interaction with TCA with the *C. difficile* bile acid germination receptor; bile acid analogues, such as CamSA (2; Figure 2) that act in this way have shown promise in pre-clinical models [73,96]. Although CamSA showed potent activity against both *C. difficile* strains 630 and VPI 10463 [73,96], it is ineffective toward the hypervirulent *C. difficile* strain R20291. In a follow-up study, Sharma et al. discovered that compound (3; Figure 2) is a potent inhibitor of *C. difficile* R20291 spore germination [97].

A further route through which bile acids interact with their host is as endogenous ligands for host cell receptor systems, including the nuclear receptor farnesoid X receptor (FXR). Recurrent CDI patients have low levels of FXR signaling, with FMT restoring this [98]. Administration of a potent FXR agonist, obeticholic acid (4; Figure 2), to a high-fat diet CDI mouse model resulted in a reduced *C. difficile* burden, as well as reduced diarrhea and improved intestinal inflammation [99]; similarly, treatment of a mouse CDI model with the tertiary bile acid, ursodeoxycholic acid (UDCA), resulted in increased FXR-related transcripts and associated reduced intestinal inflammation [100].

A further class of microbial metabolites of interest are short chain fatty acids (SCFAs), which are derived primarily from bacterial fermentation of partially and non-digestive carbohydrates within the colon. Recent studies demonstrated a deficiency in SCFAs in the GI tract in CDI, and their restoration to pre-morbid levels via FMT [89,91,101]; *in vitro* experiments highlighted close negative correlations between levels of the five carbon SCFA valerate and growth of a range of *C. difficile* ribotypes [101]. While the volatile nature of SCFAs and their potent odor presents challenges to their direct oral delivery, the administration of glycerol trivalerate (with valerate liberated in the small intestine through pancreatic lipases) was associated with reduced *C. difficile* burden in a mouse model [101]. Medium- and longer-chain fatty acids may have even more potent inhibitory effects against *C. difficile* [102,103] and these also hold promise (either via dietary manipulation, or exogenous administration) as anti-CDI agents.

Successful FMT for recurrent CDI has also been associated with changes in a number of other gut microbial metabolites [83], although whether these are purely incidental changes or hold scope for exploitation as novel targeted treatment approaches is currently undefined. Conversely, a range of other gut microbial metabolites have been recently associated with maintenance of gut epithelial integrity (e.g. tryptophan metabolites) [104], but it is currently poorly explored as to whether these metabolite systems hold relevance to CDI.

## 3. Host-directed therapeutics

Recent insights into host–pathogen interactions, pathogenesis, inflammatory pathways, and the host’s innate and adaptive immune responses are leading to identification and development of host-directed therapeutics with different mechanisms of action [105]. Emerging evidence suggests that host-directed therapeutic approaches may find utility in treating severe and refractory CDI.

### 3.1. VEGF-A

Once such potential host target is vascular endothelial growth factor A (VEGF-A), which seems to be important in promoting *C. difficile* pathogenesis. Toxigenic *C. difficile* is associated with increased colonic vascular permeability in CDI mice and elevated levels of VEGF-A *in vivo* and in human colonic mucosa exposed to toxins *ex vivo*. Both toxins A and B induced VEGF-A production in human colonocytes through HIF-alpha, p38-MAPK and MEK1/2 signaling pathways. By blocking the main receptor of VEGF-A (VEGFR-2) with a kinase inhibitor SU1498 (5; Figure 2), there was a significant reduction of vascular permeability in CDI mice, and significantly attenuated weight loss. Similarly, treatment of CDI mice with anti-VEGF-A antibody attenuated vascular permeability and protected mice from infection and improving overall survival. It is thus surmised that humanized anti-VEGF-A monoclonal antibodies such as Avastin (Bevacizumab) and its biosimilar Mvasi, which have been approved to treat various cancers and eye diseases, could prove useful drugs in managing CDI [106].

### 3.2. Intestinal ion transporters

Several intestinal ion transporters, which ensure fluid and electrolyte homeostasis, are implicated in infectious and inflammatory diarrhea [107] and may represent promising host-directed therapeutic targets against *C. difficile*.

In *C. difficile*, toxin B (TcdB) causes a pronounced inhibition of Na^+^/H^+^ exchanger 3 (NHE3) activity in placental and renal cell lines. Here, TcdB exposure inhibits NHE3 by dephosphorylation and redistribution of ezrin, which normally anchors NHE3 to the cytoskeleton [108]. Furthermore, patients with rCDI have decreased NHE3 in the apical membranes of their enterocytes [109], which contribute to the diarrheal phenotype of CDI. Injection of *C. difficile* and CDI patient stool supernatant resulted in a substantial decrease in NHE3 mRNA and protein levels compared with broth-injected (control) human intestinal organoids. *C. difficile* toxin inhibition of NHE3 alters the intestinal environment producing a high [Na^+^], and a more alkaline fluid, which enhances *C. difficile* proliferation and inhibits competitive Clostridial groups proliferation. It follows that upregulation of NHE3 may provide a means to reestablish intestinal homeostasis and thus shift the microbiota toward a pre-morbid or pre-CDI composition [109].

The down-regulated in adenoma (DRA) protein, encoded by *SLC26A3*, a key Cl^−^/HCO_3_^−^ exchanger protein in the intestinal epithelial luminal membrane, participates in electroneutral NaCl absorption, together with Na^+^/H^+^ exchangers such as NHE3 [110]. Coffing et al. demonstrated that the expression of Slc26a3 protein was significantly reduced in response to *C. difficile* toxins TcdA and TcdB in Caco 2 intestinal epithelial cells. However, these toxin-mediated effects were specific to DRA, as NHE3 and Slc26a6 protein and mRNA levels were unaffected. This discrepancy with what has been reported by Engevik et al may be due to variations in the model system and/or cell type used in addition to the different effects of *C. difficile* strains and varying concentrations of toxins [111]. Additionally, purified TcdA and TcdA and TcdB, but not TcdB alone, resulted in a decrease in colonic DRA protein levels in a toxigenic mouse model of CDI. Moreover, patients with rCDI exhibited a significant loss in colonic Slc26a3 protein, indicating that the downregulation of DRA by *C. difficile* toxins is likely occurring at the post-transcriptional level.

These results suggest that DRA and NHE3 may be potent novel targets for therapeutic management of CDI-associated diarrhea [110]. In this regard, the probiotic *L acidophilus* has been shown to increase mRNA for DRA in colonic epithelial cells by transcriptional activation of its promoter [112]. Similarly, lysophosphatidic acid (LPA) stimulates NHE3 trafficking to the apical membrane and its activity, inhibits Cl^−^/OH^−^ exchange, and both expression of and apical abundance of DRA in Caco-2 cells [113,114]. NHE3 activation by LPA is attributable to the LPA5 receptor and was abolished in the absence of NHERF2 [115,116]. LPA also increases DRA promoter activity, which involves the LPA2 receptor and the phos-phatidylinosiyol 3-kinase/AKT serine/threonine kinase 1 (PI3K/AKT) pathway [113]. Hence, these results suggest that LPA, or agents that target its receptor, may be efficacious as anti-diarrheal agents [107].

### 3.3. MicroRNAs

MicroRNAs (miRs) are small, non-coding RNAs consisting of 16 to 22 nucleotide sequences that are implicated in the regulation of gene expression by repressing the expression of target genes by degrading or inhibiting translation of the targeted mRNA [117]. These non-coding regulators of gene expression at the post-transcriptional level have an essential role in targeting transcripts encoding proteins of intestinal tight junction (TJ) and their regulators [117]. Since a defective tight-junction barrier has been implicated in inflammatory and infectious diarrhea, including *C. difficile*, the therapeutic potential of using specific microRNAs to target the intestinal TJ barrier might be of benefit.

Recent work from our own group has identified the upregulation of 64 miRNAs in the circulation of patients under-going successful FMT for rCDI [118]. In a murine model of relapsing CDI, RT-qPCR analyses of sera and cecal RNA extracts demonstrated suppression of these microRNAs, an effect that was reversed by successful FMT. In mouse colon and human colonoids, TcdB mediated the suppressive effects of CDI on miRNAs. We confirmed that the downregulation of miRNAs is likely through the suppression of Drosha (an enzyme which plays a central role in microRNA biogenesis) by CDI/TcdB and is restored following FMT. We further demonstrated that that FMT-regulated miRNAs including miR-23a, miR-150, miR-26b, and miR-28 target directly the 3ʹUTR of target IL-12B, IL-18, FGF21 and TNFRSF9, respectively. Further, a combination of two specific miRNAs, miR-23a and miR-150 demonstrated cytoprotective effects against TcdB.

Altogether, restoring miRNA expression in intestinal epithelial cells should prove beneficial in improving the intestinal barrier function in CDI. Nonetheless, significant further studies are required to understand which constituents of the gut microbiota or microbiota-derived metabolites within FMT may modulate expression of miRNAs.

Moreover, despite the progress made in understanding the role of miRNAs in mediating clinical efficacy of FMT and in maintaining gut barrier function, the clinical translation of these molecules to therapy remains in its infancy. The paucity of vehicles available for safe delivery of miRNAs, and ongoing concerns for toxicity due to off-target effects, remain major obstacles to rapid clinical translation. Continued efforts to improve the challenges inherent in miRNA therapy and substantial market investment should soon see the development of patient-specific miRNA mimics or antimiRs with a goal of effective personalized therapy for multiple conditions, including infectious diseases.

### 3.4. Immunity-based targeting

Impaired innate and adaptive immune responses have been implicated in disease severity [119–123], and treatments focused on inducing immune responses relevant to *C. difficile* clearance and recovery from CDI represent novel treatment options. Three non-antibiotic FDA-approved drugs, amoxapine (an antidepressant), doxapram (a breathing stimulant), and trifluoperazine (an antipsychotic), were demonstrated to significantly protect against experimental CDI in a mouse model by reducing bacterial burden and toxin levels. RNA-seq data indicated that these drugs can promote disease alleviation through increased expression of several innate immune response-related genes previously demonstrated to impact CDI, including those involved in the recruitment of neutrophils, the production of interleukin 33 (IL-33), and the IL-22 signaling pathway [124]. While the specific mechanism of action is unknown, experimental data suggest that the use of innate immunity to combat *C. difficile* occurs indirectly via the gut microbiota, and IL-33-mediated disease alleviation was dependent on the presence of the microbiota [124].

## 4. Drug re-purposing strategies

### 4.1. Auranofin

Due to the critical and unmet need for developing new anti-CDI therapeutics, researchers have turned their attention to repurposing drugs with well-studied safety and pharmacokinetic profiles. Auranofin (6; Figure 2) is a gold-containing anti-inflammatory FDA-approved anti-rheumatoid arthritis drug which possesses strong antibacterial and antifungal activities [125]. Similar to the anticlostridial antibiotic fidaxomicin, auranofin was observed to inhibit *C. difficile* growth, toxin production, and spore formation. Auranofin also produced a direct protective effect against *C. difficile* toxin-mediated inflammation in an *in vitro* assay, which was not observed with fidaxomicin [125,126]. Upon testing in a mouse model of CDI, auranofin significantly protected mice against CDI at low clinically achievable doses (0.125 mg/kg and 0.25 mg/kg) with 100% and 80% survival, respectively Auranofin is a promising candidate, with an unknown mechanism of action, which warrants further investigation as a novel treatment option for CDI.

### 4.2. Diiodohydroxyquinoline

Anti-parasitic drugs have also been investigated as anti-clostridial drugs. Diiodohydroxyquinoline (DIHQ, 7; Figure 2) is an FDA-approved oral anti-amoebic drug which has demonstrated potent activity against 39 *C. difficile* isolates, displaying superior efficacy to vancomycin and metronidazole in inhibiting toxin production and spore formation, whilst preserving the growth of key species within the intestinal microbiota, such as *Bacteroides, Bifidobacterium*, and *Lactobacillus* spp [127]. The mechanism of action is currently unknown.

### 4.3. Ronidazole

Ronidazole (8; Figure 2) is a veterinary antiprotozoal drug which like DIHQ can inhibit the growth of clinical *C. difficile* isolates, whilst preserving the growth of several commensal organisms in the human intestine. Ronidaxole was superior to metronidazole when both were tested at a dose of 1 mg/kg daily in a mouse model of CDI [128]. Like metronidazole, ronidazole is a member of the antibiotic class of nitroimidazole [128]. Hence, it is anticipated that the nitro group is reduced by the clostridial pyruvate:ferredoxin oxidoreductase to produce imidazole radical and nitrite, both of which are capable of causing damage to the bacterial DNA leading to cell death.

Several other potent anti-clostridial hits, clustered within four chemical groups [(nitroimidazoles MIC_50_ = 0.06–2.7 μM), salicylanilides (MIC_50_ = 0.2–0.6 μM), imidazole antifungals (MIC_50_ = 4.8–11.6 μM), and miscellaneous group (MIC_50_ = 0.4–22.2 μM) have been identified among FDA-approved drugs and are summarized else-where [128].

## 5. Natural products

Several food-grade and plant-derived natural products show demonstrable antimicrobial activity against *C. difficile* in vitro. These have been summarized by Roshan et al. [129,130]. The most active pure compounds against *C. difficile* toxins included zingerone (0.3 mg/mL), and trans-cinnamaldehyde (0.005% v/v). Three *Leptospermermum* honeys (4% w/v), and fresh onion bulb extract (12.5% v/v) also significantly reduced toxin production and activity *in vitro* [129].

Berberine (9; Figure 2) is a poorly absorbed isoquinoline alkaloid present in numerous plants of the genera *Berberis* and *Coptis*. In China, berberine has been used as a herbal medicine to treat gastrointestinal disorders for millenia [131]. It possesses a range of pharmacological and biological activities, including anti-inflammatory and antimicrobial properties against bacteria, fungi, parasites, worms, and viruses. The significant antimicrobial function of berberine is mediated through its ability to inhibit the assembly of FtsZ (filamenting temperature-sensitive mutant Z) and halt bacterial cell division [132]. Mice gavaged with berberine (100 mg/kg/day) for 5 days following standard vancomycin treatment were prevented from developing a CDI relapse in a mouse model of CDI [133]. Combined therapy prevented weight loss, improved the disease activity and histopathology scores, and effectively decreased mortality. Berberine also restored vancomycin-induced dysbiosis by inhibiting the expansion of members of the family *Enterobacteriaceae* [133].

High-throughput techniques have also been recently employed to identify new lead molecules with potent anti-clostridial activity from the AnalytiCon NATx library (consisting of 5000 natural product-inspired or natural product-derived synthetic compounds [134]. Among the panel of hits which underwent validation to confirm anti-clostridial activity, 10 compounds were found to be capable of inhibiting the pathogen. Out of these, three compounds (NAT13–338,148, NAT18–355,531, and NAT18–3,557,680) showed potent activity against *C. difficile* (MIC = 0.5–2 μg/mL), and interestingly, these compounds had minimal to no effect on the indigenous intestinal microbiota species tested and were non-toxic to a human colorectal adenocarcinoma (Caco-2) cell line [134]. Their mechanisms of action are currently unknown.

## 6. Trifluoromethylthio-containing compounds

Researchers from Purdue University, US, have recently shown how trifluoromethylthio-containing *N*-(1,3,4-oxadiazol-2-yl) benzamides altered with halogen substitutions can protect mice from CDI recurrence more effectively than vancomycin [135]. The most promising of these compounds HSGN-218 (10; Figure 2) demonstrated ultrapotent activities against various *C. difficile* isolates. HSGN-218 is up to 100 times more active [MICs ranging from 0 to 003 μg/mL (0.007 μM)] against *C. difficile* clinical isolates than vancomycin, appears to be non-toxic to mammalian colon cells, and is gut-restrictive as demonstrated by its limited ability to permeate across Caco-2 bilayers [135]. Although the precise mechanism of action of HSGN-218 is unknown, the compound belongs to the class of *N*-(1,3,4-oxadiazol-2-yl)benzamide which is known to inhibit the biosynthesis of bacterial lipoteichoic acid [136].

Further details regarding inhibitory activity for a selection of the aforementioned compounds can be found in Table 1.

## 7. Conclusions

Combating *C. difficile* continues to be a priority. Advances in molecular biology, biochemistry, and immune phenotyping, as well as high-throughput omics-based platforms have opened up a new treasure trove of therapeutic targets (Figure 2). Choosing amongst these and fine-tuning them based on a knowledge of mechanism of action and host genotypes may result in important new adjunctive therapies that are highly targeted and effective, and crucially may be indispensable in overcoming antimicrobial resistance.

## 8. Expert opinion

Despite significant efforts in recent years to improve the therapeutic armentarium against C. *difficile*, there remains a major lack of translational bench to bedside success stories. A major barrier is our incomplete understanding of C. *difficile* pathogenesis. Beyond patient characteristics and C. *difficile* ribotype or toxin production, there has been increasing recognition of the nuanced host–pathogen interaction leading to colonization and infection as well as determining disease severity and outcome. An understanding of these intricate interactions can be further accelerated through a systems biology approach to the study of CDI, including the use of high throughput ‘omic’ techniques such as next generation microbial sequencing and immunometabolic assays. How to best analyze and integrate such complex datasets arising from these techniques will require new machine-learning approaches, the development and implementation of which, will continue to challenge the research community. Recognizing the importance of host–microbiome interactions in CDI has also resulted in a shift in treatment strategy. While prevention of growth or killing of vegetative C. *difficile* cells remains important, restoring a disrupted gut microbiome may be even more critical to treatment success. Such insights into the contribution of the gut microbiome and our ability to manipulate the gut microbiome will drive further research in the next 5–10 years.

From a pharmacy and antimicrobial stewardship perspective, C. *difficile* is concerning because treatment options are limited to three antibiotics and there is also the high risk of recurrence. Academic research will continue to play a key role in identifying new drug targets, including understanding target biology but considerable hurdles remain, such as target-safety issues, druggability and assayability, as well as legal, intellectual property, and regulatory issues, all of which are important considerations for academia-industry collaborations. Future intensive efforts must focus on elucidating molecular and pathophysiological mechanisms that underpin susceptibility to primary and recurrent infection and the most severe manifestations of C. *difficile*-associated disease. To address this need, more time and effort should be invested in prioritizing the development of gut infection model systems with greater relevance to human disease, such as patient-derived primary cells or induced pluripotent stem cells. These approaches can complement animal and three-tier chemostat models of CDI. Greater emphasis on supporting the immune system to neutralize C. *difficile* by disarming the synthesis of virulence or pathogenesis factors, and thereby reducing evolutionary pressure for the emergence of resistance, may offer the best approach. Accordingly, drugs targeting certain virulence factors, such as adhesins or toxins, as well as biofilm or spore formation, represent ideal candidates for further intensive exploration. Moreover, host directed therapeutics are likely to gain traction as prospective adjunctive therapies, which modulate the inflammatory response and restrict immunopathology. However, concerns remain regarding delivery systems and off-target effects. Whilst C. *difficile* biofilm communities are likely critical in rCDI, several questions remain unanswered regarding the relevance of such biofilm communities in bacterial persistence and the bacterial and host factors regulating their formation in vivo. In terms of toxin inhibitors, it should be borne in mind that the PaLoc Island comprising the main toxins, TcdA and TcdB, are very variable in their composition resulting in multiple toxinotypes. Moreover, the high plasticity of the C. *difficile* genome is another important challenge for the rationale design of future therapies. Thus, implementing whole-genome sequencing combined with metabolomics approaches, and emerging high-plex digital spatial and molecular profiling technologies will facilitate an unprecedented level of subcellular resolution. These advanced ‘omics’ tools, coupled with machine learning and artificial intelligence methods, will provide better ‘deep’ phenotyping of clinical trial patients, and will help elucidate targets and pathways critical to those processes amenable to pharmacologic modulation. Artificial intelligence can also be used to identify at-risk patients sooner and afford clinicians the opportunity to address modifiable risk factors, thereby proactively preventing CDI using known, evidence-based prevention strategies.

As the cost of developing a new drug remains prohibitively expensive, repurposing of existing approved and investigational drugs such as auranofin, diidohydroxyquinoline and ronidazole may quickly open orthogonal approaches, particularly given known safety profiles and reduction in the cost barrier.

Overall, the implementation of a multidisciplinary team science and an inter-organizational collaborative model will be required to foster innovation and facilitate efficient drug discovery programs. Although target identification is critical to developing new effective compounds for the treatment and prevention of CDI, it is likely that no magic bullet model for CDI is attainable and that only a combination of targets will mediate a full phenotypic effect.

## Figures and Tables

**Table 1.** Emerging anti-*C. difficile* therapeutics: inhibitory activity, dose, usage and benefits.

	Drug/treatment	Inhibitory activity	Dose	Usage	Benefits
Repurposed drugs	Amoxapine	MIC> >100 μg/ml – no bactericidal activity	3 mg/kg, i.p. daily for 3 days	Repurposed -an antidepressant – non-antibiotic FDA-approved drug [124]	Drug-mediated protection was not due to bacterial killing or modulation of toxin production *in vivo* and has limited impact on the microbiome
	Doxapram	MIC> >100 μg/ml – no bactericidal activity	20 mg/kg, i.p. daily for 3 days	Repurposed – a breathing stimulant – non-antibiotic FDA-approved drug [124]	
	Trifluoroperazine	MIC> >100 μg/ml – no bactericidal activity	1.5 mg/kg, i.p. (single dose)	Repurposed – an antipsychotic – non-antibiotic FDA-approved drug [124]	
	Auranofin	MIC90 = 0.5 μg/ml	0.125 mg/kg and 0.25 mg/kg (mice)	Repurposed – FDA-approved anti-rheumatoid arthritis drug which posseses strong antibacterial and antifungal activities [125].	Inhibits toxin production, spore formation and protects human gut cells against the inflammation induced by *C. difficile* toxins
	Diiodohydroxyquinoline	MIC_50_ = 0.5 μg/ml	650 mg/kg – TID 20 days (for amoebiasis)	Repurposed – FDA-approved oral anti-amoebic drug which has potent activity against *C. difficile* [127]	Superior to Vancomycin and fidaxomycin at inhibiting toxin production and spore formation and did not inhibit the growth of key gut species
	Ronidazole	MIC = 0.0625 μM	1 mg/kg daily	Repurposed – Veterinary antiprotozoal drug which like DIHQ can inhibit the growth of clinical *C. difficile* isolates [112]	Superior to metronidazole in a mouse model of CDI at same dose
	Nitroimidazoles	MIC50 = 0.06–2.7 μM	n/a	Repurposed – potent anti-clostridial hits [128]	Not yet tested in animals
	Salicylanilides	MIC_50_ = 0.2–0.6 μM	n/a		
	Imidazole antifungals	MIC50 = 4.8–11.6 μM	n/a		
	Halicin	MIC = 0.5 μg/ml	200 mg/kg i.p. in mice single daily dose 3 days)		Greater clearance of *C. difficile* in mice than metronidazole
	Miscellaneous group	MIC50 = 0.4–22.2 μM	n/a		Not yet tested in animals
Natural products	Berberine	MIC vary from 1.024–256 μg/ml	100 mg/kg/day for 5 days Post Vancomycin	Prevented relapse post vancomycin in a mouse model of CDI [133], isoquinoline alkaloid present in numerous plants, but its not effective against all isolates	Prevented weight loss, improved the disease activity and histopathology scores, expansion of *Enterobacteriaceae*
	NAT13-338,148, NAT18-355,531, and NAT18-3,557,680	MIC = 0.5–2 μg/mL	n/a	AnalytiCon NATx library – natural product library [134]	Minimal to no effect on the indigenous intestinal microbiota species
Trifluoromethylthio-containing compounds	Trifluoromethylthio-containing N-(1,3,4-oxadiazol-2-yl) benzamides (HSGN-218)	MIC > 0.007 μM	50 mg/kg/day for 5 days (mice)	Ultrapotent small molecule inhibitor of various isolates and protects mice from rCDI [135]	Upto 100 times more active than vancomycin, it inhibits *C. difficile* growth and is non-toxic to human cells. It has a moderate effect on the microbiota.
FabK targets	Verapamil	MIC50 = 8 μM	n/a	Targets bacterial fatty acid synthesis pathway [70]	Narrow spectrum
	Verapamil + efflux inhibitors	MIC50 = 2 μM	n/a		More potent with the addtion of efflux inhibitors

QID = four times daily; TID = three times daily; TID = three times daily; DIHQ = Diiodohydroxyquinoline; MIC = Minimum inhibitory concentration; i.p. = intraperitoneal

## Article highlights

- An increase in treatment failures and recurrence rates has intensified the search for novel therapeutic targets for the development of next-generation therapeutics for the prevention and treatment of CDI
- Knowledge on new molecular therapeutic targets and early drug candidates is emerging from improved understanding of *C. difficile* biology and host–microbe interactions
- Drug target identification may also benefit from investigating the effects of epigenetic regulators on disease processes
- The most promising newly emerging therapeutic targets for clinical development include drugs targeting the toxins, adhesins, biofilms, spores, *p*-cresol biosynthetic pathway, fatty acid synthesis protein enoyl-acyl carrier protein reductase II (FabK) and potentially, gut microbiota-derived metabolites.
- The most promising host-directed novel therapeutic targets include those targeting the receptor for vascular endothelial growth factor A (VEGF-A), innate immunity, intestinal ion channels, and microRNA-based therapeutics which regulate inflammation and gut barrier function.
- Prioritization of lead targets for those most amenable to therapeutic intervention should be undertake after extensive *in vitro* and *in vivo* testing and validation work across multiple assay systems and organotypically relevant disease models
- Developing solutions for preclinical biological data sharing and accessibility and incentivising multidisciplinary collaborations may allow the field to rapidly progress
